# Retraction: Mitochondrial tRNA mutations associated with essential hypertension: From molecular genetics to function

**DOI:** 10.3389/fcell.2022.1006531

**Published:** 2022-08-04

**Authors:** Frontiers Editorial Office

The Publisher retracts the cited article.

Following publication, the publisher uncovered evidence that false identities were used in the peer-review process. The assignment of a fake reviewer was confirmed by an investigation, conducted in accordance with Frontiers’ policies and the Committee on Publication Ethics (COPE) guidelines.

Because the peer-review had been compromised, the investigation included a post-publication review of the article, which concluded that the article should have been rejected because it does not meet the standards for publication in Frontiers in Cell and Developmental Biology.

The authors do not agree to this retraction.

This retraction was approved by the Chief Editors of Frontiers in Cell and Developmental Biology and the Chief Executive Editor of Frontiers.

